# Exploring the virulence potential of *Staphylococcus aureus* CC121 and CC152 lineages related to paediatric community-acquired bacteraemia in Manhiça, Mozambique

**DOI:** 10.1038/s41598-024-61345-3

**Published:** 2024-05-10

**Authors:** Marcelino Garrine, Mariana Andrade, Joana Neves, Inácio Mandomando, Isabel Couto, Sofia Santos Costa

**Affiliations:** 1 Centro de Investigação em Saúde de Manhiça (CISM), Maputo, Mozambique; 2 Global Health and Tropical Medicine, GHTM, Associate Laboratory in Translation and Innovation Towards Global Health, LA-REAL, Instituto de Higiene e Medicina Tropical, IHMT, Universidade NOVA de Lisboa, UNL, Rua da Junqueira 100, 1349-008 Lisbon, Portugal; 3 Instituto Nacional de Saúde (INS), Ministério da Saúde, Maputo, Mozambique; 4 ISGlobal-Hospital Clínic, Universitat de Barcelona, Barcelona, Spain

**Keywords:** Microbiology, Bacteriology, Biofilms, Pathogens

## Abstract

*Staphylococcus aureus* is a frequent agent of bacteraemia. This bacterium has a variety of virulence traits that allow the establishment and maintenance of infection. This study explored the virulence profile of *S. aureus* strains causing paediatric bacteraemia (SAB) in Manhiça district, Mozambique. We analysed 336 *S. aureus* strains isolated from blood cultures of children younger than 5 years admitted to the Manhiça District Hospital between 2001 and 2019, previously characterized for antibiotic susceptibility and clonality. The strains virulence potential was evaluated by PCR detection of the Panton-Valentine leucocidin (PVL) encoding genes, *lukS-PV/lukF-PV*, assessment of the capacity for biofilm formation and pathogenicity assays in *Galleria mellonella*. The overall carriage of PVL-encoding genes was over 40%, although reaching ~ 70 to 100% in the last years (2014 to 2019), potentially linked to the emergence of CC152 lineage. Strong biofilm production was a frequent trait of CC152 strains. Representative CC152 and CC121 strains showed higher virulence potential in the *G. mellonella* model when compared to reference strains, with variations within and between CCs. Our results highlight the importance of monitoring the emergent CC152-MSSA-PVL^+^ and other lineages, as they display important virulence traits that may negatively impact the management of SAB paediatric patients in Manhiça district, Mozambique.

## Introduction

*Staphylococcus aureus* is one of the major causes of bacteraemia and other infections in humans^1^. The versatility of this pathogen is partly determined by a varying repertoire of virulence factors, including toxins that increase the pathogen potential to cause a range of diseases^2^. Clinically important toxins encoded by *S. aureus* comprise the toxic shock syndrome toxin-1 (TSST-1), staphylococcal enterotoxins (SEs), exfoliative toxins (ETs), and the Panton-Valentine leucocidin (PVL), encoded by the *lukS-PV/lukF-PV* genes^2,3^. PVL is a bi-component exotoxin that forms pores in the membrane of leukocytes, inducing their lysis^4^. Although carriage of the *lukS-PV/lukF-PV* genes has been related to specific types of infections, such as skin and soft tissue infections and necrotizing pneumonia, the role of PVL in bacteraemia is still controverse^5,6^.

Biofilm production is another important virulence determinant in *S. aureus*^7^. Overall, biofilm producing strains are estimated to be associated with 65–80% of all bacterial infections in humans^8^. The biofilm formation is a complex process, in which several components participate, such as the polysaccharide intercellular adhesin (PIA), encoded by the *ica* operon, and *S. aureus* surface protein G (encoded by *sasG* gene)^7^. Those infections are notorious for their chronicity and resilience against therapy, with a consequent increase in morbidity and mortality^7^. Therefore, a better understanding of staphylococcal biofilms is imperative to generate new treatment strategies for biofilm-associated infections, and to reduce their significant impact on disease.

Despite the importance of PVL and biofilm on the *S. aureus* pathogenesis, most of the studies are based on the epidemiology of the disease, on antimicrobial susceptibility determination, or screening of genes encoding specific resistance determinants and virulence factors, with scarce information regarding the in vitro or in vivo virulence potential of important circulating strains. Several studies evaluated the virulence potential of clinical *S. aureus*, but only a few were based on infection models that generate in vivo data quickly and inexpensively, such as the *Galleria mellonella* larval infection model^9–13^. The increasing use of this model is supported by its innate immune response which is similar to the one of mammals, including similar mechanisms of pathogen killing^13,14^. However, most of these reports determining the virulence potential of *S. aureus* strains are from high and middle-income countries^9,11,12^. As the circulating *S. aureus* clonal lineages differ from region to region, studies determining the virulence potential of the main clonal lineages circulating in Africa are needed, particularly those associated with severe disease (e.g. bacteraemia). We recently reported the epidemiology and clinical characteristics of children with *S. aureus* bacteraemia (SAB) over two decades (2001–2019) in the Manhiça district, Mozambique^15^. Afterwards, the SAB-related *S. aureus* strains (N = 336) were analysed for their clonality, antibiotic resistance phenotypes and resistance determinants^16^. The data gathered throughout the two decades of the study period revealed an evolution in the predominant clonal lineages over time. Particularly, we observed a predominance of methicillin-susceptible *S. aureus* (MSSA)-clonal complex (CC)152 and MSSA-CC121 in the last six years of the study (2014–2019); in contrast to the predominance of CC1, CC5, CC8, CC15, CC25, CC80 and CC88 between 2001 and 2013.

The present study aimed to evaluate the virulence profile of the CC121 and CC152 *S. aureus* related to SAB in our setting. The strains virulence profile was characterized by screening the genes encoding for PVL toxin, the biofilm-associated genes *icaADB*, *atlA* and *sasG*, and evaluating the strains capacity to produce biofilms, whereas the virulence potential of representative strains was assessed in the *G. mellonella* larval infection model.

## Results

### Frequency of *lukS-PV*/*lukF-PV* genes among SAB-related *S. aureus* strains

**Figure 1:**
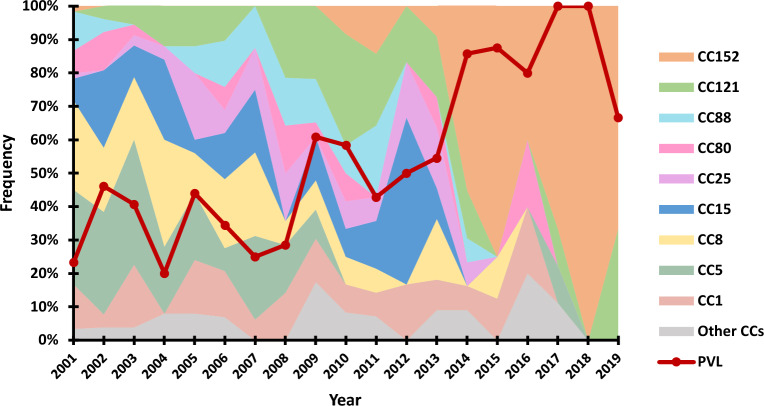
Relation between the frequency of carriage of *lukS-PV/lukF-PV* genes encoding the Panton-Valentine leucocidin (PVL) and *S. aureus* clonal complexes (CCs) detected at the Manhiça District Hospital throughout the study period. The number of total *S. aureus* isolates per year of the study period are as follows (2001–2019)^15^: 60, 26, 32, 25, 25, 29, 16, 14, 23, 12, 14, 6, 11, 14, 8, 5, 9, 4 and 3.

Overall, the *lukS-PV/lukF-PV* genes were detected in 43.7% (147/336) of the *S. aureus* strains. The comparative analysis of the presence of *lukS-PV/lukF-PV* genes and the MLST CCs previously described for this collection^16^ revealed predominance of these genes among strains belonging to CC22 (100%), CC152 (96.9%) and CC121 (85.3%), followed by CC88 (69.2%), CC80 (62.5%) and CC1 (47.2%) (Table 1). The frequency of *lukS-PV/lukF-PV* genes among the remaining CCs varied between 0 and 40%. Analysing the variation in the frequency of detection of *lukS-PV/lukF-PV* genes throughout the study period, we could observe two distinct trends (Fig. 1). During the first twelve years of the study (2001–2013), the frequency of PVL-positive strains varied between ~ 20 and ~ 60%. In the following years (2014–2019), we observed an increasing trend in PVL-positive strains, accounting from ~ 70% up to 100% of all SAB-related *S. aureus* strains isolated in that timeframe. Of particular interest, the increasing trend of PVL was linked to the emergence of the CC152 strains.

**Table 1:** Relation between frequency of carriage of *lukS-PV/lukF-PV* genes and antibiotic resistance phenotypes among the *S. aureus* clonal lineages detected at the Manhiça District Hospital.

Clonal complex (CC) ^a^	Sequence type (ST) ^a^	*lukS-PV/lukF-PV* genes, n/N (%)	MRSA^a^, n/N (%)	MDR^a^ n/N (%)
CC121 (N = 34)	ST121, ST2430, T6102	29/34 (85.3)	0 (0)	9/34 (26.5)
CC152 (N = 33)	ST152	32/33 (96.9)	0 (0)	12/33 (36.4)
CC5 (N = 58)	ST5, ST650, ST6	23/58 (39.6)	0 (0)	6/58 (10.3)
CC8 (N = 56)	ST8, ST612, ST72	2/56 (3.6)	15/56 (26.7)	22/56 (39.3)
CC15 (N = 37)	ST15, ST1160, ST1906	1/37 (2.7)	0 (0)	2/37 (5.4)
CC1 (N = 36)	ST1	17/36 (47.2)	0 (0)	9/36 (25.0)
CC88 (N = 26)	ST88	18/26 (69.2)	1/26 (3.8)	6/26 (23.1)
CC80 (N = 16)	ST80, ST6994	10/16 (62.5)	0 (0)	1/16 (6.2)
CC25 (N = 20)	ST25	7/20 (35.0)	0 (0)	16/20 (80.0)
CC22 (N = 6)	ST22	6/6 (100.0)	0 (0)	1/6 (16.7)
CC45 (N = 8)	ST508	2/8 (25.0)	0 (0)	1/8 (12.5)
CC12 (N = 3)	ST12	0 (0)	0 (0)	0 (0)
CC97 (N = 1)	ST97	0 (0)	0 (0)	0 (0)
Singletons (N = 2)	ST3502, ST7349	0 (0)	0 (0)	0 (0)

*MRSA* methicillin-resistant *S. aureus*, *MDR* multidrug-resistant *S. aureus.*

^a^Data from Garrine et al*.*, 2023b^16^.

Similar frequencies of *lukS-PV/lukF-PV* genes were observed among multidrug resistant (MDR) and non-MDR strains (42.3%, 36/85 vs. 44.2%, 111/251, respectively; *p* = 0.764). Additionally, the *lukS-PV/lukF-PV* genes were not detected among the methicillin-resistant *S. aureus* (MRSA) isolates. Expanding this analysis to include relevant clinical data (length of stay (LOS) and mortality)^15^, no statistically significant difference was observed between LOS of patients infected by *S. aureus* strains either carrying or not the *lukS-PV/lukF-PV* genes (4 days, IQR: 3–7 vs. 5 days, IQR: 3–8, respectively; *p* = 0.370). No statistical association was also observed between *lukS-PV/lukF-PV* genes carriage and mortality (28.0%, 7/25 vs. 43.7%, 111/254, respectively; *p* = 0.129).

### Biofilm production and screening of biofilm-associated genes

**Figure 2:**
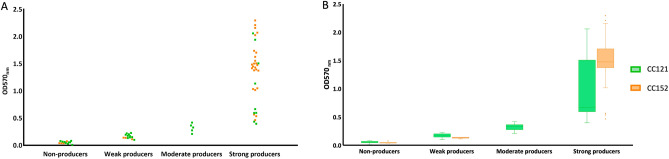
Distribution of CC152 and CC121 strains according to biofilm production phenotype (**A**); variation and median OD570_nm_ values (**B**).

Overall, the capacity to produce biofilms was observed for 80.0% (52/65) of the CC121 and CC152 strains tested. The frequency of biofilm producing strains (weak, moderate or strong producers) was similar between CC152 and CC121 strains (87.5%, 28/32 vs. 72.7%, 24/33, respectively; *p* = 0.137). However, considering the level of biofilm production, CC152 strains were significantly more often classified as strong producers than CC121 strains (78.1%, 25/32 vs. 27.3%, 9/33, respectively; *p* < 0.0001) (Fig. 2A). This observation extends to the median OD570_nm_ values observed for the two groups of strains, particularly when comparing strong biofilm producers (Fig. 2B).

In general, the biofilm production phenotype was not related to MDR phenotypes for either of the clonal lineages studied, even when they were analysed in combination [CC121 + CC152] or separately [CC121 (77.8%, 7/9 MDR vs. 70.8%, 17/24 non-MDR, *p* = 1.000); CC152 (83.3%, 10/12 MDR vs. 90.0%, 18/20 non-MDR, *p* = 0.620)]. Similarly, infection by biofilm-producing strains was not related to extended LOS compared to non-producing strains (5 days, IQR: 2–7 vs. 4 days, IQR: 3–5, respectively; *p* = 0.712). Also, no association was observed when the LOS was analysed separately for CC121 (5.5 days, IQR: 3–7.5 for biofilm-producing strains vs. 4 days, IQR: 3–4 for non-producing strains, *p* = 0.464)) and CC152 (4 days, IQR: 2–7 for biofilm-producing strains vs. 5 days, IQR: 3–7 for non-producing strains, *p* = 0.859). The frequency of *lukS-PV/lukF-PV* genes was similar between biofilm-producing and non-producing strains (92.3%, 48/52 among biofilm producers vs. 84.6%, 11/13 among non-biofilm producers, *p* = 0.591), even when analysed in separate for CC121 (87.5%, 21/24 among biofilm producers vs. 77.8%, 7/9 among non-biofilm producers, *p* = 0.597) and CC152 (96.4%, 27/28 among biofilm producers vs. 100%, 4/4 among non-biofilm producers, *p* = 1.000).

The screening of biofilm-associated genes showed that *icaADB*, part of the *ica* operon, were present in all CC121 and CC152 strains tested, including non- and strong-biofilm producers. The *atlA* gene, encoding a major staphylococcal autolysin was present in 98.5% (64/65) of strains tested. The single strain that did not carry this gene was a CC121 biofilm strong producer. The *sasG* gene was absent from most strains, with an overall frequency of 7.7% (5/65), corresponding to two CC121 (5.9%, 2/34) and three CC152 (9.1%, 3/33) strains. Overall, these results indicate that the presence or absence of these genes in CC121 and CC152 is not directly related to the biofilm producing phenotype observed.

### Virulence potential of representative CC121 and CC152 *S. aureus* strains in the *G. mellonella* infection model

**Figure 3:**
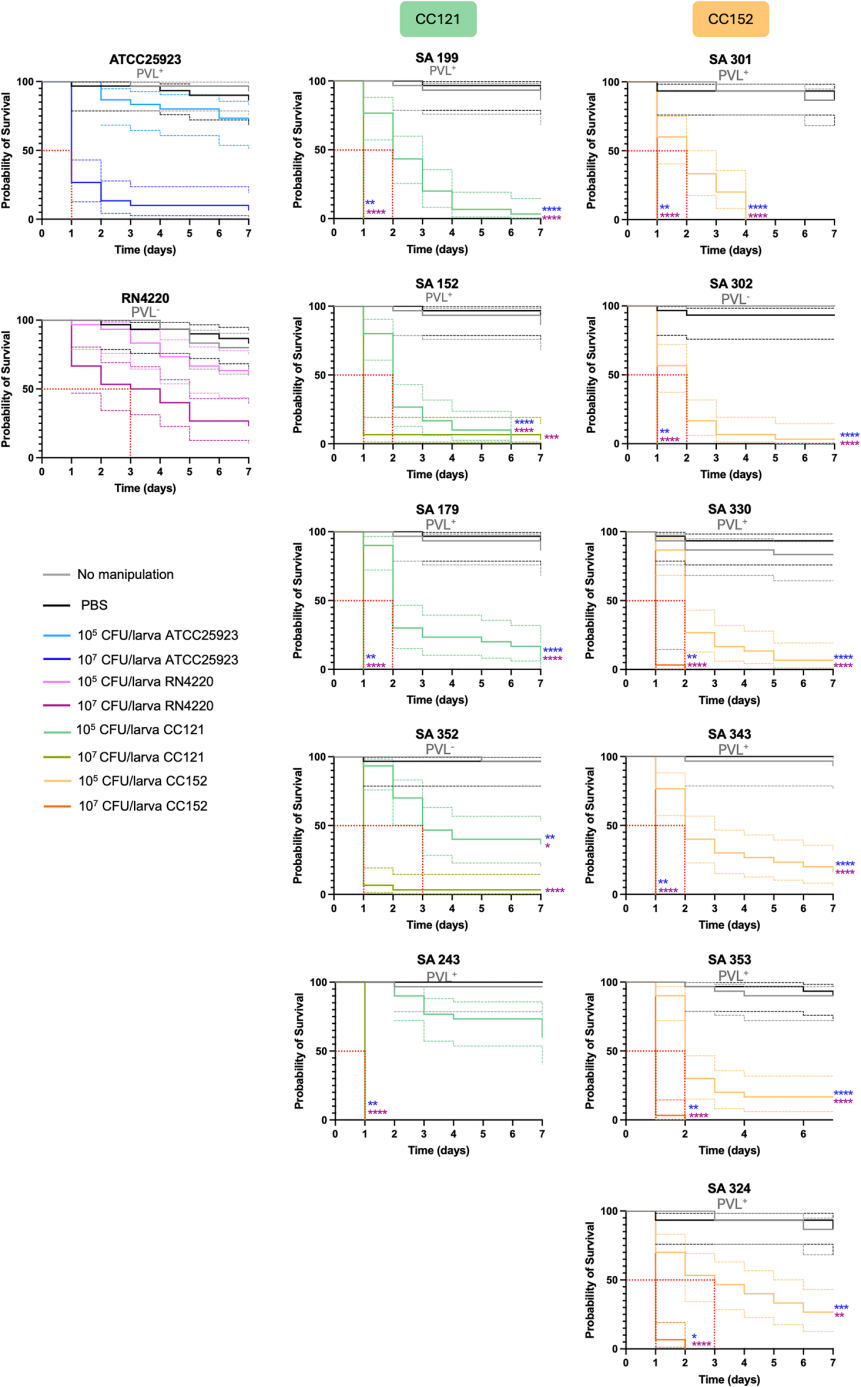
Kaplan–Meier survival analysis of *G. mellonella* infection assays with *S. aureus* ATCC25923 (in blue), *S. aureus* RN4220 (in purple) and SAB-related *S. aureus* strains representative of clonal lineage CC121 (in green) and CC152 (in orange). The dotted lines indicate the 95% confidence intervals and the red dashed lines indicate the median survival time. Statistically significant differences between each strain and the reference strains *S. aureus* ATCC25923 and RN4220 (at corresponding inoculums) are identified as follows: *p < 0.05, **p < 0.01, ***p < 0.001 and ****p < 0.0001.

The virulence potential of eleven representative strains of the emergent lineages circulating in our setting, CC121 and CC152, was assessed in the larval *G. mellonella* infection model. Overall, all clinical strains tested showed high virulence in *G. mellonella* when compared to the reference strains *S. aureus* ATCC^®^25923™ and *S. aureus* RN4220. Analysing the survival curves and median survival time, we observed a variation of the virulence potential within and between the CCs analysed. Comparing CC121 strains, SA 199 (PVL^+^) was the most virulent, followed by SA 152 (PVL^+^) > SA 179 (PVL^+^) > SA 352 (PVL^−^) > SA 243 (PVL^+^) (Fig. 3). Similarly, CC152 strains could be differentiated regarding their virulence potential as follows, SA 301 (PVL^+^) > SA 302 (PVL^−^) > SA 330 (PVL^+^) > SA 343(PVL^+^) > SA 353 (PVL^+^) > SA 324 (PVL^+^). For all strains, the median survival time was inversely proportional to the bacterial inoculum; from 2 to > 7 days at 10^5^ CFU/larva to only 1 day at 10^7^ CFU/larva (Table 2). In agreement with our findings, previous reports described mortality of *G. mellonella* larvae infected with *S. aureus* to be dose-dependent, in which infection with 1 × 10^7^ CFU/larva resulted in death after 24 h, whereas infection with 1 × 10^5^ CFU resulted in ∼20% of deaths after 120h^17^.

**Table 2:** Main virulence and antibiotic resistance traits of representative SAB-related *S. aureus* strains selected for the assays in the *G. mellonella* infection model.

Strain ID	Clonal Complex (CC), (ST)^a^	PVL encoding genes	Biofilm production phenotype	Mean OD_570_ value	Median survival time (days) and survival-associated *p*-value				Resistance profile^a^	Patients outcome^b^
					10^5^ inoculum	*p*-value vs. 10^5^ ATCC25923/RN4220	10^7^ inoculum	*p*-value vs. 10^7^ ATCC25923/RN4220		
ATCC 25923	CC30 (ST243)	+	Strong producer	2.134	> 7	–	1	–	–	–
RN 4220	CC8 (ST8)	−	Strong producer	1.271	> 7	–	3.5	–	–	–
SA 152	CC121 (ST121)	+	Strong producer	1.134	2	** < 0.0001**/< **0.0001**	1	0.2426/**0.0002**	Non-MDR	Survived
SA 179	CC121 (ST121)	+	Non-producer	0.030	2	** < 0.0001**/< **0.0001**	1	**0.0026/ < 0.0001**	Non-MDR	Died
SA 199	CC121 (nd)	+	Moderate producer	0.209	2	** < 0.0001**/< **0.0001**	1	** < 0.0001**/ < **0.0001**	MDR	Died
SA 243	CC121 (nd)	+	Weak producer	0.144	> 7	0.4843/0.9683	1	**0.0026/ < 0.0001**	MDR	Survived
SA 352	CC121 (ST121)	−	Non-producer	0.061	3	**0.0063/0.0286**	1	0.1350**/< 0.0001**	MDR	Survived
SA 301	CC152 (ST152)	+	Non-producer	0.037	2	** < 0.0001**/< **0.0001**	1	**0.0026/< 0.0001**	Non-MDR	Survived
SA 302	CC152 (ST52)	−	Strong producer	1.431	2	** < 0.0001**/< **0.0001**	1	**0.0026/< 0.0001**	MDR	Survived
SA 324	CC152 (ST152)	+	Strong producer	2.024	3	**0.0002/0.0230**	1	**0.0172/< 0.0001**	Fully susceptible^c^	Survived
SA 330	CC152 (ST152)	+	Strong producer	2.158	2	** < 0.0001**/ < **0.0001**	1	**0.0077/< 0.0001**	MDR	Survived
SA 343	CC152 (nd)	+	Non-producer	0.038	2	** < 0.0001**/ < **0.0001**	1	**0.0026/< 0.0001**	Non-MDR	Died
SA 353	CC152 (ST152)	+	Strong producer	0.535	2	** < 0.0001**/ < **0.0001**	1	**0.0077/< 0.0001**	MDR	Survived

^a^Data from Garrine et al*.* 2023b^16^.

^b^Data from Garrine et al*.*, 2023a^15^.

^c^Fully susceptible to the tested antibiotics (Garrine et al*.* 2023b)^16^.

*ST* sequence type, *MDR* multidrug-resistant *S. aureus* (defined as those strains not susceptible to three or more unrelated classes of antibiotic).

Significant differences are highlighted at bold type. *nd* not determined.

When analysing the overall virulence potential of *S. aureus* CC121 and CC152 lineages, no relation was found with the virulence traits studied (carriage of *lukS-PV/lukF-PV*, biofilm-associated genes or biofilm production phenotype) or with the patient outcome, i.e. higher virulence potential did not correlate with poorer patient outcome, and vice-versa (Table 2).

We also analysed the virulence potential of an additional set of four strains, representative of CC8 clonal lineage. Although present in lower frequency, this lineage comprises the majority (15 out of 16) of the MRSA strains detected in our setting over the two decades of the study period (Table 1). Similarly to the other CCs analysed, the CC8 strains (ST8 and ST612) showed a virulence potential higher than the reference strains. Within this clonal complex, the ST8 strains (SA 215, MSSA; SA 277, MRSA) were the most virulent, compared to the two ST612-MRSA strains, SA 276 and SA 188 (Supplementary Fig. S1 and Supplementary Table S1). In general, the CC8 strains showed similar virulence potential to the CC121 and CC152 strains.

## Discussion

We recently reported the high diversity of *S. aureus* clonal lineages from children with bacteraemia admitted to the Manhiça District Hospital (MDH), Mozambique with predominance of the CC121 and CC152 (emergent clone) in the last years of the study period, using the surveillance system established in this setting^16^. These same clones were recently detected circulating in other African countries^18^, suggesting their important role in the epidemiology of staphylococcal invasive infections in this region.

This is one of the few studies assessing the virulence potential of SAB-related *S. aureus* in African countries. Despite the absent correlation between the bacterial virulence traits analysed and the median survival time of *G. mellonella*, a higher virulence potential was established for the SAB-related *S. aureus* strains circulating in Manhiça District, compared to reference strains, particularly for strains from the emergent CC152 lineage. The same pattern of higher virulence potential was detected for other CC8 clinical strains from our collection (Supplementary Fig. S1 and Table S1). Several virulence factors carried in *S. aureus* are often encoded on the pathogen’s accessory genome. The clinically most important toxins encoded by *S. aureus* are SEs, TSST-1, ETs and PVL, responsible for necrotic lesions involving skin and mucosa, and necrotic haemorrhagic pneumonia^2,3^. Almost half (43.7%) of the entire *S. aureus* study collection carried the PVL determinants *lukS-PV/lukF-PV* and these predominated among specific clonal lineages. In our study, these genes predominated among CC121, CC152 and CC22 lineages, supporting the observation of previous reports^18–21^. The increase of PVL in the last years of the surveillance period was most likely due to the increase of the CC152-MSSA-PVL^+^, although some caution should be taken in this analysis, considering the low rates of *S. aureus* isolated in this period. The absence of a relation between biofilm production, presence of biofilm-associated genes (*icaADB*, *atlA*, *sasG*), PVL encoding genes (*lukS-PV/lukF-PV*), resistance profile and the virulence of either CC121 or CC152 strains in *G. mellonella* infection model, suggests the contribution of additional virulence factors to the overall virulence potential. Noteworthy, in our study, *lukS-PV/lukF-PV* genes predominated from both MDR and non-MDR strains and were absent among MRSA strains. We have previously found that infection by these MDR strains was associated to higher mortality of SAB patients in our setting^15^. In the present study, infection of *G. mellonella* by MDR strains did not result in a significantly different survival time when compared with infection by non-MDR strains.

Our results revealed a high virulence potential of the CC152 lineage predominantly circulating in Manhiça in the last years of the study^16^. This finding is matter of concern since CC152 has been reported as an important emergent and prevalent clone in several African countries^18,22^. CC121, for which we have also established a high virulence potential, is a clonal lineage also increasingly identified in the African continent^18,23^. Further detailed epidemiological and molecular analysis of these important clones is urgently needed for prompt adoption of adequate prevention and control measures.

## Conclusions

To our knowledge, this is one of the first studies documenting the virulence potential of SAB-related *S. aureus* from Mozambique. Here we report a high frequency of MSSA but not MRSA strains carrying the determinants for PVL toxin. Strains from the lineage CC152 and, in a lesser extent, CC121, that have established in our setting in recent years are frequent strong biofilm producers and show higher virulence potential in the *G. mellonella* infection model when compared to two prototype reference strains. Although no correlation could be established between particular clonal lineages and virulence, the approach described in this study has potential to be applied to large datasets in future studies.

Our results highlight the importance of monitoring the emergent CC152-MSSA-PVL^+^ and other lineages as they display important virulence traits that may impact negatively the management of SAB paediatric patients in Manhiça, Mozambique.

## Methodology

### Study design and *S. aureus* collection

In this study we analysed *S. aureus* strains isolated from blood cultures of children admitted to the Manhiça District Hospital (MDH, Manhiça District, southern Mozambique) between 2001 and 2019. The pertinent characteristics of our *S. aureus* collection (N = 336), previously described^15,16^, are presented in Table 1. These strains are part of the 24h morbidity surveillance ongoing in MDH as one of the main activities of the *“Centro de Investigação em Saúde de Manhiça”* (CISM). The surveillance performs a standardized collection of clinical data among all paediatric patients (< 15 years of age) and a specific microbiological surveillance based on the systematic collection of blood cultures among admitted patients, according to the relevant guidelines and regulations^15,24^.

### PCR screening of genes encoding PVL and biofilm-associated genes

All isolates were cultivated in blood agar plates, followed by overnight incubation at 37 °C. Afterwards, one colony was transferred to 5 mL of Tryptic Soy Broth (TSB, Oxoid Ltd., Basigstoke, UK), and incubated overnight at 37 ºC with constant shaking (180 rpm). Total DNA was extracted by the boiling method according to an established protocol^25^. The three hundred and thirty-six SAB-related *S. aureus* strains were screened by conventional PCR for the presence of a 433 bp internal fragment of the *lukS-PV/lukF-PV* genes, encoding PVL, using the primers luk-PV-1 (5′-ATCATTAGGTAAAATGTCTGGACATGATCCA-3′) and luk-PV-2 (5′-GCATCAASTGTATTGGATAGCAAAAGC-3′) and conditions previously established^26^. The sets of thirty-four CC121 and thirty-three CC152 strains were further screened for the presence of *atlA*, *sasG* and *ica* genes. A 359 bp fragment comprising the 3′-terminal region of *icaA*, the entire *icaD* gene and the 5′-terminal region of *icaB*, was amplified using the primers icaADB_SA_Fw (5′-AGTTCTTGTCGCATTTCCAA-3′) and icaADB_SA_Rv (5′-CACGATTCTCTT CCTCTCTGC-3′)^12^. A 621 bp internal fragment of the *altA* gene was searched using altA_SA_Fw (5′-CAGGTAAGTGGACAGATGCT-3′) and altA_SA_Rv (5′-GGATGTCGAAGTATTTGCCG-3′). For the *sasG* gene, a 598 bp fragment was amplified using sasG_SA_Fw (5′-CGTTCTGTTGATGAAGGCTC-3′) and sasG_SA_Rv (5′-GTTGCCCATGAAACTTTCCA-3′).

### Evaluation of biofilm production by CC121 and CC152 strains

We evaluated the biofilm mass production for most strains from CC121 (97.1%, 33/34) and CC152 (97.0%, 32/33). The strains capacity for biofilm production was assessed by the crystal violet adhesion method^27^, using the conditions described elsewhere^12^. Briefly, the isolates were cultured in TSB medium and incubated overnight without shaking at 37 °C. A cellular suspension adjusted to ~ 5 × 10^8^ CFU/mL in TSB was prepared, diluted 1:100 in TSB supplemented with 1% glucose and 3% NaCl and 0.2 mL aliquots distributed in PS flat-bottom TC 96 well microtiter plates (Orange Scientific, Braine-L’ Alleud, Belgium), followed by incubation at 37 °C during 24 h. Afterward, the wells content was discarded and washed with PBS. Adherent bacteria were fixed with methanol (99%) for 20 min and air-dried overnight. The biofilm mass was dyed with crystal violet (0.1%) for 15 min, washed with distilled water and air-dried. The crystal violet was solubilized in acetic acid (33%) for 30 min and the optical density at 570_ nm_ (OD570) was measured in a Synergy HT microplate reader (Biotek, Winooski, VT, USA). Each assay included in quadruplicates, the control strains *S. aureus* ATCC^®^25923™ (moderate to strong biofilm producer), *S. epidermidis* ATCC^®^12228™ (weak to moderate biofilm producer, *ica*^-^), and *S. epidermidis* ATCC^®^35984™ (strong biofilm producer, *ica*^+^), as well as the *S. aureus* strains under testing, a negative control (supplemented TSB), and a blank (33% acetic acid). Biofilm production was also assessed for reference strain *S. aureus* RN4220 (moderate to strong producer). Biofilm production was categorized according to Stepanović's criteria^27,28^, which establishes a cut-off value (ODc) defined as the geometric mean of OD570 of the negative control (supplemented TSB) + 3 × the corresponding standard deviation (SD). Strains were characterized as follows: OD_strain_ < ODc, biofilm non-producers; ODc < OD_strain_ < 2 × ODc, weak producers; 2 × ODc < OD_strain_ < 4 × ODc, moderate producers; OD_strain_ > 4 × ODc, strong producers. Each assay was performed, at least, in duplicate. To minimize intra- and inter-assay variability, an assay was only validated when associated with an SD value < 20% of the corresponding geometric mean (either considering an individual assay or duplicates) and assigning the strain to the same category. A strain was considered a biofilm producer if assigned to the weak, moderate or strong producer phenotypes.

### Assessment of the virulence potential of *S. aureus* in the *G. mellonella* infection model

The virulence potential was evaluated for eleven representative strains of CC121 and CC152, selected according to the presence/absence of PVL encoding genes, potential of biofilm production, resistance profile (MDR/non-MDR) and outcome of infected patients (Table 2). The *G. mellonella* were reared at GHTM/IHMT-NOVA at 28 °C (± 1 °C) under dark with a high nutrient diet (corn flour, dried yeast, soy flour, dry milk, honey, glycerol, bee wax blocks). Larvae at the final instar stage, weighting 300 mg ± 50 mg and with no signs of melanisation were used in the infection assays. We determined the virulence potential of each strain using two distinct bacterial inoculums, 10^5^ and 10^7^ CFU/larva. The procedures for bacterial inoculum preparation and larvae infection were conducted as described elsewhere^12^. Briefly, bacteria were inoculated in TSB and incubated overnight at 37 °C with agitation. The culture was washed in PBS, resuspended and adjusted to ~ 5 × 10^8^ CFU/mL, then diluted in 1:100 PBS to achieve ~ 5 × 10^6^ CFU/mL and ~ 5 × 10^4^ CFU/mL, respectively. Bacteria enumeration was determined by CFU counting. Each assay included four groups of *G. mellonella* larvae (n = 10 larvae/group), as follows: (i) non-manipulated; (ii) inoculated with PBS (control); (iii) inoculated with 10^5^ CFU/larva; (iv) inoculated with 10^7^ CFU/larva. All larvae were inoculated with a 0.02 mL inoculum by injection with an insulin syringe (Omnican 100, Braun) in the last proleg. The *G. mellonella* survival was monitored each 24 h post-infection for seven days, as previously described^14^. The virulence potential of each staphylococcal strain was evaluated in, at least, three independent infection assays^12^.

### Data analysis

Data from virulence assays in the *G. mellonella* infection model were analysed by Kaplan–Meier survival curves using GraphPad Prism v8.0.1 (San Diego, CA, USA). Survival rates between groups were compared with the Log-Rank (Mantel-Cox) test. Data on *S. aureus* antibiotic resistance phenotype (MDR/non-MDR; MRSA/MSSA) and clonal lineages^16^ as well as the patients outcome (hospital discharge)^15^ were compared with virulence traits (*lukS-PV*^+^*/lukF-PV*^+^, carriage of *icaADB*, *atlA* or *sasG* genes and biofilm production phenotype) using χ^2^ or Fisher’s exact test as appropriate (STATA version 14.2, StataCorp LP, College Station, Texas, USA). Wilcoxon rank sum tests were used for nonparametric comparisons (e.g. patients Length of stay, LOS). We deemed a *p-*value of 0.05 or lower to be statistically significant.

### Ethical approval

The *S. aureus* isolates analysed in this study fall within the scope of the ongoing morbidity and microbiological surveillance system established as part of the Health and Demographic Surveillance System at CISM, approved by the Institutional Ethics Review Board for Health at CISM and the National Bioethics Committee for Health. All residents of Manhiça district have signed individual informed consent forms to participate in the ongoing surveillance.

### Supplementary Information

Supplementary Information.

## Data Availability

All relevant data have been provided in the paper. Raw data are available from the corresponding author on reasonable request.
