# Supplementary material for: Exploring the virulence potential of Staphylococcus aureus CC121 and CC152 lineages related to paediatric community-acquired bacteraemia in Manhiça, Mozambique
Source: Sci Rep. 2024 May 10;14:10758. doi: 10.1038/s41598-024-61345-3 (PMC11087594; doi:10.1038/s41598-024-61345-3)
Supplement: Supplementary file 1 — Supplementary Information. [file 41598_2024_61345_MOESM1_ESM.docx]

**Exploring the virulence potential of *Staphylococcus aureus* CC121 and CC152 lineages related to paediatric community-acquired bacteraemia in Manhiça, Mozambique**

^
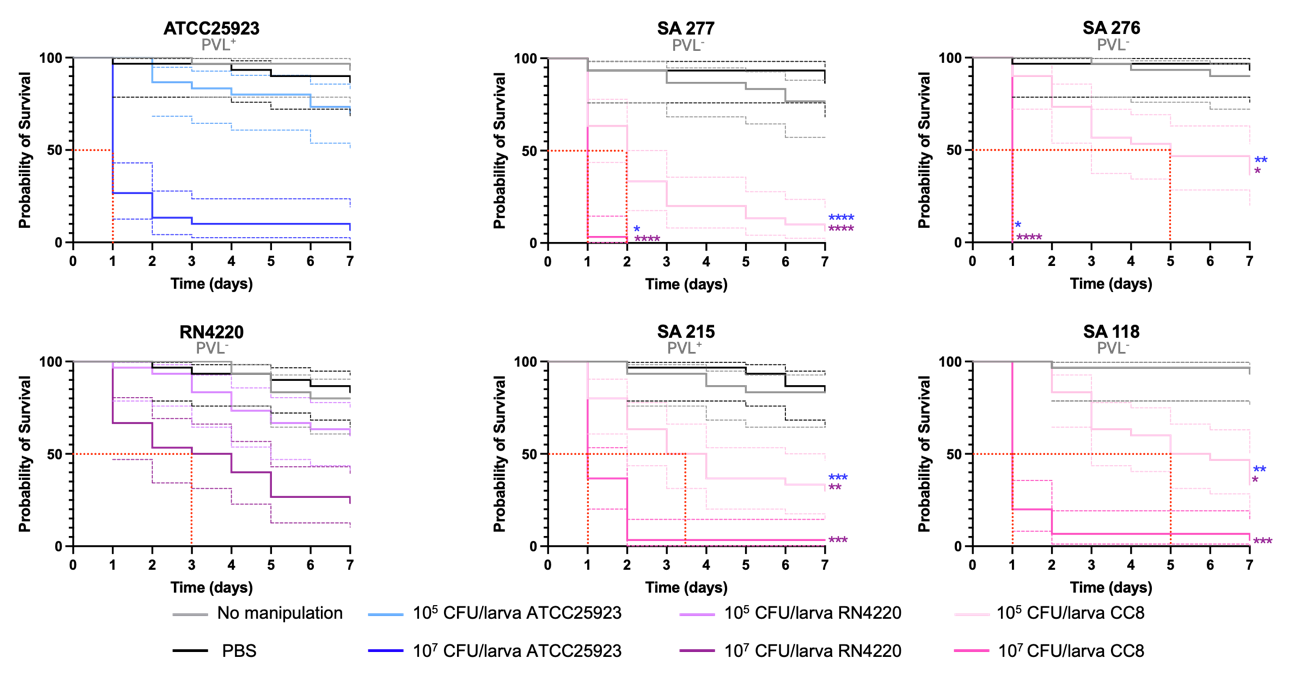
^Marcelino Garrine, Mariana Andrade, Joana Neves, Inácio Mandomando, Isabel Couto, Sofia Santos Costa

**Supplementary Figure S1.** Kaplan-Meier survival analysis of *G. mellonella* infection assays with *S. aureus* ATCC25923 (in blue), *S. aureus* RN4220 (in purple) and SAB-related *S. aureus* strains representative of clonal lineage CC8 (in pink). The dotted lines indicate the 95% confidence intervals and the red dashed lines indicate the median survival time. Statistically significant differences between each strain and the reference strains *S. aureus* ATCC25923 and *S. aureus* RN4220 (at corresponding inoculums) are identified as follows: * *p* < 0.05 and ** *p* < 0.01.

**Supplementary Table S1. Main virulence and antibiotic resistance traits of representative SAB-related *S. aureus* strains of the CC8 clonal lineage selected for additional assays in the *Galleria mellonella* infection model.**

| **Strain ID** | **Clonal Complex (CC), (ST)^a^** | **PVL encoding *lukSF-PV* genes** | **Biofilm production phenotype** | **Mean OD_570_ value** | **Median survival time (days) and *p*-value** | | | | **Resistance profile^a^** | **Patients outcome^b^** |
| --- | --- | --- | --- | --- | --- | --- | --- | --- | --- | --- |
|  |  |  |  |  | **10^5^ inoculum** | ***p*-value vs. 10^5^ATCC25923/RN4220** | **10^7^ inoculum** | ***p*-value vs. 10^7^ATCC25923/RN4220** |  |  |
| SA 118 | CC8 (ST612) | - | Moderate Producer | 0.196 | 5 | **0.0070** / **0.0487** | 1 | 0.4836 / **0.0003** | MDR | Died |
| SA 215 | CC8 (ST8) | + | Non-Producer | 0.062 | 3.5 | **0.0009** / **0.0063** | 1 | 0.9002 / **0.0002** | Non-MDR | Survived |
| SA 276 | CC8 (ST612) | - | Strong Producer | 0.410 | 5 | **0.0084** / **0.0491** | 1 | **0.0375** / **<0.0001** | MDR | Survived |
| SA 277 | CC8 (ST8) | - | Strong Producer | 2.159 | 2 | **<0.0001** / **<0.0001** | 1 | **0.0375** / **<0.0001** | MDR | Survived |

^a^Data from Garrine *et al.*, 2023b;^16^ ^b^Data from Garrine *et al.*, 2023a;^15^ ST, sequence type; MDR, multidrug resistant *S. aureus* (defined as those strains not susceptible to three or more unrelated classes of antibiotic). Significant differences are highlighted at bold type.
